# Health-related expectations of the chronically critically ill: a multi-perspective qualitative study

**DOI:** 10.1186/s12904-020-00696-w

**Published:** 2021-01-04

**Authors:** A. Fuchsia Howard, Sarah Crowe, Laura Choroszewski, Joe Kovatch, Adrianne Jansen Haynes, Joan Ford, Scott Beck, Gregory J. Haljan

**Affiliations:** 1 School of Nursing, The University of British Columbia, T201 – 2211 Wesbrook Mall, Vancouver, BC V6T 2B5 Canada; 2 Fraser Health Authority, Surrey, British Columbia Canada; 3 Patient Partner, Vancouver, British Columbia Canada; 4 Faculty of Medicine, The University of British Columbia, Vancouver, British Columbia Canada

**Keywords:** Chronic illness, Critical illness, Chronic critical illness, Respiration, Long term mechanical ventilation, Qualitative, Health services, Quality of life, Palliative care

## Abstract

**Background:**

Those who survive critical illness only to become chronically critically ill (CCI) experience a high symptom burden, repeat episodes of illness exacerbation, communication barriers, and poor health outcomes. Yet, it is unclear how CCI individuals and their family understand their health and the importance of prognostic information following hospitalization. The research purpose was to examine expectations about health and disease prognosis of CCI residents in long-term care from the perspectives of the CCI themselves and their family members, as well as to describe healthcare provider (HCP) interpretations of, and reactions to, these health-related expectations.

**Methods:**

In this qualitative interpretive descriptive study, conducted in British Columbia, Canada, 38 semi-structured interviews were conducted (6 CCI residents, 11 family members, and 21 HCPs) and inductively analyzed using thematic and constant comparative techniques.

**Results:**

There was divergence in CCI resident, family and HCP expectations about health and the importance of disease prognosis, which contributed to conflict. CCI residents and family viewed conflict with HCPs in relation to their day-to-day care needs, while HCPs viewed this as arising from the unrealistically high expectations of residents and family. The CCI residents and family focussed on the importance of maintaining hope, and the HCPs highlighted the complexity of end-of-life decisions in conjunction with the high expectations and hopes of family.

**Conclusions:**

The emotional and ongoing process of formulating health-related expectations points to the need for future research to inform the development and/or adapting of existing communication, psychosocial and health services interventions to ease the burden experienced by those who are CCI.

**Supplementary Information:**

The online version contains supplementary material available at 10.1186/s12904-020-00696-w.

## Background

There is an emerging population of patients who survive their critical illness owing to treatment advances in the intensive care unit (ICU), but who do not fully recover and instead progress to persistent dependence on life-sustaining treatment. Though definitions vary, prolonged dependence on mechanical ventilation after an acute episode of critical care is a hallmark of chronic critical illness, which is frequently accompanied by metabolic, immunological, neuroendocrine, neuromuscular, cognitive and psychological disturbances, and repeat episodes of infection and organ dysfunction [1, 2]. The burden of chronic critical illness is enormous and survival grim, with roughly half of patients dead at 1 year and only 1 in 5 eventually discharged home [3–6]. In the United States, chronically critically ill (CCI) individuals are often cared for in facilities termed long-term acute care hospitals, which are among the fastest growing segments of the healthcare system [7]. Globally, several models of care have emerged, including dedicated weaning centres [8], respiratory care centres or units [9], nurse-led special care units [10], and specific care programs in a traditional ICU [11, 12]. In Canada, where long-term acute care hospitals do not exist, the CCI are increasingly discharged to long-term care facilities capable of providing ventilator care.

Prior research, largely taking place close to the time of transition to chronic critical illness (often based on tracheostomy and need for prolonged mechanical ventilation), suggests poor quality of life and high symptom burden among patients, high family anxiety and distress, and overly optimistic health expectations of family [13–15]. Similar findings were described in a recent study in an American long-term acute care hospital wherein nearly 80% of patient and their surrogate decision makers identified going home as a goal, while only 38% were home at 1 year [16]. There is also growing evidence of discordance in prognostication of patient outcomes, including survival and regaining of functional independence and cognitive states, between surrogate decision makers and healthcare providers - physicians and nurses [14, 15, 17]. There are suggestions that surrogate decision makers’ optimistic expectations are potentially modifiable [15]. Increasingly, there are recommendations to design and test interventions that improve patient and family understanding of prognosis [14], and yet, the complexity of health-related expectations and the role these expectations play in the context of CCI individuals’ lives following acute care remains largely unknown. As such, there is uncertainty about the best ways to intervene to support surrogate decision makers or CCI patients [18], particularly nearing end-of-life.

Despite the emerging research, there is insufficient evidence to fully inform the care of CCI patients [19]. Once discharged from acute care, patient and family expectations about health and prognosis are perhaps re-considered and re-formulated as time goes on, in light of evolving, vacillating and declining health status as well as the institutions where patients reside. Anecdotally, CCI patients, family members and healthcare providers (HCPs) are routinely faced with the on-going challenge of making decisions about treatment and goals of care, especially with the onset of new illness, exacerbation of chronic disease or near end-of-life. However, it is unclear how patients and family understand the health and wellbeing of the CCI individual, the importance or priority of prognostic information and how this plays out in long-term care. Patient-perspective evidence articulating CCI patients’ experiences and perspectives is vital to patient-centred interventions and models of care for this unique and rapidly expanding population [20]. This is particularly salient considering concerns and debate about whether CCI patients receive more life-prolonging treatment than appropriate. The purpose of this research was to examine expectations about health and disease prognosis of CCI residents in long-term care from the perspectives of the CCI themselves and their family members, as well as to describe healthcare provider (HCP) interpretations of, and reactions to, these health-related expectations.

## Methods

Our team of researchers, clinicians, healthcare managers and administrators, and patient and family partners designed and conducted this patient-oriented [21], qualitative interpretive description [22] research. Our team included two critical care clinicians, a nurse practitioner and a physician who provide outreach to the practice setting; three administrators from the practice setting; two patient and family partners with related lived experience though not in the specific clinical setting; a nurse researcher and a nurse graduate student research assistant.

### Setting and sample

This research was conducted in a 22-bed specialized unit in a long-term care facility in Canada where CCI ventilated patients reside, and which represents the second largest cohort of CCI individuals in the province. In Canada, there is a public health care system that provides universal health care inclusive of residential care for CCI individuals. We invited all CCI residents and a family member designated by the residents to participate, as well as the surrogate decision maker for patients who were not cognitively intact or who were unable to be understood by the interviewer via an assistive communication device. Inclusion criteria for the CCI resident participants included being cognitively intact (as indicated by the attending physician), English speaking and ventilator dependent but able to communicate verbally, by mouthing words, blinking and nodding or through use of adaptive technology. Family members were included if either a resident consented to the research team to invite them to participate, or they were the surrogate decision maker for a resident because the resident was not cognitively intact or could not communicate verbally. Further inclusion criteria for family members included being cognitively intact and English speaking. We also invited all of the 45 HCPs employed on the specialized unit to participate. We interviewed all residents, family and HCPs who consented to being contacted by the research team and to participating in the study, and then used purposive sampling to recruit participants with diverse characteristics. Because a member of the resident’s circle of care team initially approached potential residents and family members to obtain consent for the research team to invite them to participate in the study, we are unaware of how many individuals were approached and declined. Though 8 residents initially consented to being contacted by the research team, one declined participation stating it was too upsetting for her to think about the subject matter, and another passed away.

### Data collection

We conducted semi-structured interviews with CCI residents, family and HCPs, using interview guides that built on Lamas et al. [23], conversation guide for chronic critical illness to query health-related expectations. An additional file contains the interview guides (see Additional File 1). However, these interview guides served as a means of facilitating conversation and we encouraged participants to communicate their experiences and perspectives as they wished and asked additional questions that emerged throughout the dialogue. As is typical of qualitative interpretive description data collection, we did not adhere strictly to the interview guides, but rather, the interviewers followed the participant’s lead by encouraging elaboration on aspects of their experiences related to their health and disease prognosis expectations. Interviews with CCI residents and family were conducted at the resident’s bedside, in a separate conference room at the facility, or off-site at a community centre, according to participant preference, and lasted 45–120 min. These interviews were either conducted independently or in conjunction with family, depending on resident preference and two interviewers were present to facilitate interpretation of resident communication. Following the resident and family interviews, we collected resident demographic and medical information from the medical chart.

As per their expressed preference, interviews with HCP were conducted via telephone rather than at the facility to maintain participant confidentiality, with interviews ranging from 30 to 45 min long. All study interviews were audio-recorded and transcribed verbatim. Of note, we only report minimum participant demographic and resident medical information in order to ensure anonymity. The interviews with residents and family were overwhelmingly rich in information power [22], owing to interviews lasting much longer than anticipated at the preference of participants, participants sharing highly emotional, detailed and personal narratives, variation in perspectives and experiences shared, and the qualitative and clinical expertise of the interviewers. With resident, family and HCP participation, we were confident that we obtained sufficiently high information power and variation in participant characteristics for a rigorous interpretive description study.

### Data analysis

Data collection and preliminary analysis occurred in an iterative manner, where we asked additional questions in subsequent interviews as a way of elaborating on earlier interviews. We began our thematic analysis and developed our initial coding frame by highlighting transcript segments that reflected emerging patterns, diversities and examples of resident, family and HCP accounts. We revised the coding frame based on extensive research team discussions and proceeded to code all interview data using the data management software, NVivo™ [17]. We then used constant comparative methods to compare pieces of data, and we regrouped analytic codes into broader categories, reflecting higher levels of conceptualization [22, 24, 25]. When comparing and contrasting resident and family interview data, there was substantial convergence. However, there was marked divergence when comparing and contrasting resident/family data to the HCP data accounts. Finally, we refined the analytical categories into themes that represent an interpretive description of health-related expectations of CCI individuals and family, with HCP perspectives also included. The convergence and divergence of participant data is represented in the grouping and reporting of resident and family accounts together, and separate from those of HCPs. Research team members, inclusive of researchers, clinicians, administrators, and patient and family partners, were involved extensively throughout the data collection and analysis of interview data, which was supported through regular group meetings typified by open dialogue and discussion.

## Results

A total of 38 individuals participated in this research; 6 CCI residents, 11 family members, and 21 HCPs. The 6 residents and 11 family members represented a total of 12 CCI individuals who resided in the facility (2 residents completed an interview independently, 4 residents completed a dyad interview with a family member, and 7 family members completed an interview independently). Five of the 7 family members who completed an interview independently did so because the resident was either not cognitively intact or had severely impaired communication abilities, 1 of the 7 did so at the resident’s request for separate interviews, and the resident recently passed away in 1 of the 7 cases. See Table 1 for a breakdown of the study participants who participated in individual and dyad interviews and Table 2 for participant demographic information and disease characteristics.

**Table 1:** Participants by Type, Interview and Corresponding Family Participant

Participant #	Participant Type	Interview Type (Individual or Dyad)	Corresponding Resident/Family Participant	Resident Characteristics Represented in Table 2
**1**	**Resident**	**Individual**	**Family did not participate**	**Yes**
**2**	**Resident**	**Individual**	**Participant #13**	**Yes**
**3**	**Resident**	**Dyad**	**Participant #14**	**Yes**
**4**	**Resident**	**Dyad**	**Participant #15**	**Yes**
**5**	**Resident**	**Dyad**	**Participant #16**	**Yes**
**6**	**Resident**	**Dyad**	**Participant #17**	**Yes**
**7**	**Family**	**Individual**	**Resident did not participate**	**Yes**
**8**	**Family**	**Individual**	**Resident did not participate**	**Yes**
**9**	**Family**	**Individual**	**Resident did not participate**	**Yes**
**10**	**Family**	**Individual**	**Resident did not participate**	**Yes**
**11**	**Family**	**Individual**	**Resident did not participate**	**Yes**
**12**	**Family**	**Individual**	**Resident did not participate**	**Yes**
**13**	**Family**	**Individual**	**Participant #2**	**No – abstracted from Participant #2**
**14**	**Family**	**Dyad**	**Participant #3**	**No – abstracted from Participant #3**
**15**	**Family**	**Dyad**	**Participant #4**	**No – abstracted from Participant #4**
**16**	**Family**	**Dyad**	**Participant #5**	**No – abstracted from Participant #5**
**17**	**Family**	**Dyad**	**Participant #6**	**No – abstracted from Participant #6**

**Table 2:** Characteristics of Study Participants

***Residents characteristics represented by both resident and family participants***	***n*** **= 12**
**Age, years**	
20–39	2
40–59	4
60+	6
**Sex**	
Male	5
Female	7
**Cultural Background**	
Asian	3
Caucasian	7
South Asian	2
**Most recent primary ICU admission diagnosis**	
Trauma	1
Sepsis	4
Respiratory failure	5
Neurological Insult	2
**Time from Admission to Interview**	
< 1 year	6
1–5 years	4
> 5 years	2
**Advanced Care Directive**	
Cardiopulmonary Resuscitation (CPR)	7
Do Not Resuscitate (DNR)	5
**Method of Communication**	
Verbal	6
Mouthing Words	3
Blinking and Nodding	2
Eyegaze Technology	1
**Level of Function**	
Wheelchair, independent	4
Wheelchair, dependent	7
Bedridden	1
***Family Members who participated in an interview***	***n*** **= 11**
**Age, years**	
20–39	1
40–59	8
60+	2
**Sex**	
Male	2
Female	9
**Relationship to Resident**	
Parent	2
Spouse	2
Child	5
Sibling	2
***Healthcare Providers who participated in an interview***	***n*** **= 21**
**Position**	
Nurse	16
Other healthcare provider	2
Administrative	3
	**Mean**
**Length of Employment, years**	6.42
**Age, years**	32

The resident and family expectations about resident health and disease prognosis shared in this study were similar, but also diverged from those of HCPs. Conflict between residents/family and HCPs was described by participants, wherein the residents/family viewed this in relation to their day-to-day care needs, while HCPs viewed this conflict as arising from the unrealistically high expectations of residents and family. Further, the residents and family focussed on the importance of maintaining hope, yet the HCPs participants conveyed the complexity of end-of-life decisions in conjunction with the high expectations and hopes of family.

### Expectations about health and disease prognosis

#### Residents/family

Among the residents and family, there was variation in their ability to discuss what they understood to be their prognosis and disease trajectory. The majority acknowledged the resident’s poor prognosis, and most expected that they or their family member will pass away while living in the facility. For many, this knowledge was key to focussing their attention on the day-to-day rather than worrying about the future.

*I know my mom’s illness is not good, too good. She will go down. I know it will go down. This result we can’t avoid looking at. That’s why I want every day with my mom. That’s why I do not think about the future. I just do my best, so every day, is a new day. And I look at just that day. – Family Member*

Yet, the discomfort and difficulty of engaging in discussions about their health expectations was typified by those who became tearful, quiet, seemed to be at a loss for words and changed the topic. There were also residents who did not want information or to be reminded about probable health declines, as evident in the response of a family member who was asked whether they discuss with the resident their goals of care:

*No. No, he doesn’t, no. That’s, that’s something that he’s never wanted to talk about. Even really kind of before he was sick. He would just kind of ignore that. It’s just, it was an executive decision we made as a team, cause he won’t, he wouldn’t answer that question.*

Despite acknowledging a poor prognosis, the residents and family commonly depicted the future course of their illness to be uncertain, with comments such as, “it’s all a big question mark, what is going on with me?” and “I never really know how much of a future there is.” This uncertainty was bolstered by drawing on their experiences of already surviving a life-threatening condition. Some conveyed the possibility that they/their loved one could live longer than HCPs expected, enabling them to maintain hope that they could similarly beat the odds again.

*We’re kind of expecting that the end would come in the not too distant future. But we’ve been saying that for years now. Doctor [name] you know he’s been trying to prepare us for this for years. He had a chat with us about you know the, she’s in, it could be a week. And this is four years ago. And he seemed to be expecting her to go long, long, long ago. – Family Member*

Others expressed fear that they were running out of time and “could pass away any day,” and thus, were desperate to spend their remaining time engaging in meaningful activities and focused on their quality of life.

*I feel scared because I feel like I’m up against time because I know my condition, it’s not going to get any better. Most important is quality of life with the time I have. I’m not saying I want to do anything dangerous, but I want to be able to do things that might make my days a little more pleasant. - Resident*

The resident and family commentaries suggested that making sense of their likely future illness trajectory involved more than the logical interpretation of information, but rather, was coloured by emotions, ongoing loss, and the long process of grieving. As one HCP reflected, “But sometimes I find there’s just so much else, emotion and feelings, that I don’t know if they [residents] truly understand.”

#### HCPs

According to the majority of HCPs, residents and family commonly had unrealistically high expectations about the resident’s prognosis. HCPs expressed dismay and frustration with the residents and family who maintained hopes or beliefs that disease symptoms would improve over time.

*One of the things that just struck me, was they [family] asked, “is he gonna get better?” This is a resident who has [progressive disease] and from the documentation and even what the family had expressed, they had been told about [progressive disease]. But they were still holding on to, is he gonna get better? Maybe they were thinking, okay, what can [facility] do? Or maybe we have some other treatments, or maybe we have a different thinking … - HCP*

*A lot of them I think they, they don't understand. They’re kind of in denial. They are not understanding. They always think they're going to get better - HCP*

There was widespread HCP commentary on how residents avoided conversations about their probable health decline and inevitable end-of-life. They perceived that residents were aware of their diagnosis, but tended not to be able to translate this information into an understanding about stages of decline, especially from one day to the next.

*Other residents that I talk to here, they’re pretty much accepting of their diagnosis. But, I don’t think they are ready for what’s going to happen to them at the end. Cause their expectation is that they’re going to live, and live forever, and ever. The disease is not going take that toll. - HCP*

These expectations persisted despite what HCPs considered to be efforts to inform or educate families, including in-person meetings and telephone calls with various members of the healthcare team to discuss the resident’s current health state as well as goals of care. Yet, HCPs also offered the view that although residents and family were given information about their disease and prognosis, they were unable to process sometimes-devastating information and breakdowns in communication were common. It was unclear, however, whether breakdowns in communication were due to the nature of information, how it was delivered, or both. A HCP explained how seeing a resident decline was unsettling because she did not know whether the family fully understood the progression of the disease.

*I find it difficult to comment with the family. Sometimes they don't know, or they do not expect … I don't know what they’re told and … I find it hard to talk to the families. - HCP*

Importantly, means of addressing psychological needs were largely considered by HCPs to be insufficient.

### Conflict between residents/family and HCPs

#### Residents/family

According to the residents and family, conflict with HCPs largely occurred in relation to their day-to-day care needs, as described elsewhere [26], rather than their expectations about their prognosis. However, communication with HCPs related to regular health updates and the management of new and ongoing health challenges was described as “sparse,” “limited” and “just hard to get.” As such, some commented on their ongoing struggle to obtain information from HCPs, as well as constantly “pushing” and advocating for care they thought appropriate.

*We always have to ask for everything. We always had to ask for the tests and it was the same thing when we saw the new doctor. He had a bladder infection and went to the hospital, and they inserted the catheter in him, and they just leave it in, leave it in, leave it in. And we’re asking, what—why is it still in there, right? So we ask for it to take out. I just don’t understand why they’ve left it in there for a few months without saying, ‘Oh, you could get another infection,’ or this or that, so it just doesn’t make sense to me. We always have to ask for everything. You know? So many instances, you were driving the care and have to push. We have—if we don’t, you know, speak up, nothing gets done, you know? – Family Member*

Some residents and family further expressed the desire for more open, transparent, in-depth and frequent communication about ongoing health management. In sharp contrast to the HCPs, the residents and family did not discuss anger, frustration or conflict associated with health declines or end-of-life decisions.

#### HCPs

HCPs told narratives of family members lashing out in anger and frustration at times. They described being puzzled, offended, and upset by these outbursts and attributed the events to family members’ “unrealistic high expectations” of care and prognosis. Many of these conflicts arose in response to a serious degradation in the resident’s health status, and some HCPs expressed difficulty presenting information in a way that the individual could hear and accept the next phase of loss in their already limited life. HCPs described how conversations at the time of health decline were received with resistance and emotion, and “I’ll have that conversation and the anger [from the resident], right away.” At each stage of decline, HCPs might face conflict with residents and family members, leading them to believe that when residents received their diagnosis, they received little information about what to expect over time. Yet, a few HCPs interpreted the conflict between residents, family, and HCPs as a form of denial.

*Say they’re losing control over their body, but they don’t want to, they’re like, they’re [in] denial, right? They want staff to push like extra hard to do something about this. But that’s like not under control of the staff … they basically will go from one person to another and ask the same thing over and over, or get mad at you, for no reason. Their frustration is something else. - HCP*

Other HCPs focused on what they termed “challenging family members” with “way high expectations” as the most difficult aspect of their work, commenting that family members who become more demanding during health setbacks “create a negative environment … they just go with their emotions and they blame and they can’t control [themselves].” Although these family interactions were not the norm and admittedly the minority of families, the interactions appeared to leave lasting imprints on HCPS. But, many HCPs also acknowledged that “some family members are actually really, really helpful. They’ll be here every day and help us do care … they know how we are stressed …”.

A senior HCP described the importance of establishing a trusting relationship with families and stated that “lack of communication” was responsible for most conflicts. S/he said that conflicts could be resolved by more listening and communication: “I would go down and sit with the family for an hour …” This approach, however, appeared not to be the norm in the very busy and highly routinized environment. HCPs wanted residents and family to be cooperative and accept the inevitable decline inherent in the disease. They worked hard to provide good medical care, but they appeared unrealistically pragmatic about how patients should accept their medical diagnosis and prognosis despite the constant steady losses inherent in these illnesses. At the same time, some emphasized feeling ill equipped to defuse emotionally volatile situations on their own or provide the requisite emotional support to both residents and family.

### The importance of maintaining Hope

#### Residents/family

Rather than discuss their health and healthcare expectations, the residents and family emphasized the importance of hope, which entailed finding something to hope for, even if relatively small, and maintaining hope, even when their hopes shifted. Almost all of the residents and family described their hopes in relation to engaging in meaningful activities and/or spending time with loved ones, not their medical prognosis. Meaningful activities they hoped for included spending time outside the facility or in nature, going on outings to a sports event, attending spiritual or church services, or attending a family wedding or reunion. Residents and family often adjusted their hopes as their health stabilized or declined. A family member explained the value of preserving and sharing small, everyday things:

*She hasn’t seen outside this room other than to go to the hospital … My hopes now are simpler things... To be able to take her to a mall for a couple of hours. To be able to take her outside. – Family Member*

While many acknowledged that what they hoped for was unrealistic, they conveyed the impression that dreaming and imagining was imperative to carrying on with life. This kind of hope was adaptive and represented the compassion of family members.

#### HCPs

HCP participants were less prone to talk about the role of hope in helping residents cope with illness and their poor prognosis. They were more likely to express concerns that residents and family did not fully comprehend their diagnosis and the trajectory of the disease; however, at the same time they understood the emotional distress the situation exerted on family and were unsure how much reality and factual knowledge residents and family even wanted. As a result, some HCPs described feeling compelled to balance an attitude of positivity and hope with the knowledge of likely decline and associated suffering.

*You can’t take hope away, right? I think taking away hope makes people angry and defensive and more likely to think the medical system itself is not caring, not adequately supporting their loved one. So, it’s not going in there and saying, ‘we can fix this,’ it’s more not taking away hope. - HCP*

At the same time, as witnesses to the deterioration of many residents over time, HCPs carried the knowledge of what difficulties were in store for residents and family, but they lacked supports in their own role to manage these competing values: hope versus honesty. A HCP shared the sense of these competing interests, leaning towards hope:

*Some of them, I would say they don't know that they have a terminal illness. They expect, like they will be better one day. They're going to start walking and stuff. Sometimes I find it is difficult how to explain it to them, because I don't want to give them a shock. Like telling them, oh no they're not going to go home. - HCP*

### Complexity of end-of-life decisions

All residents in the facility have explicit advanced care directives, of which all HCPs are aware, that are formulated through conversations during meetings with the resident, family and care team. In regard to emergencies, directives, and end-of-life decisions, several HCP participants expressed bewilderment that some residents had instructions for full resuscitation orders.

*My opinion, as far as maybe up to about a year ago, we had a lot of full codes. Why? I don’t want to pass judgment, I mean whatever your belief is, that’s your belief and I can’t pass judgment either way, but, like wow! You can only move your eyes, why are you at full code? - HCP*

The HCPs also conveyed the complexity of end-of-life decisions in conjunction with the high expectations and hopes of family. Because some residents lose the ability to speak and communicate, the issue of who communicates and takes responsibility for the residents’ end-of-life preferences could become fraught with ethical and logistical tensions. HCPs described a type of “denial” that some family members practiced, such as refusing to believe that the individual’s speech was no longer intelligible or that their loved one would not decline any further. These participants shared narratives about family members wanting to keep their loved one alive regardless of circumstances. These “unrealistic hopes” from family members could potentially impede the wishes of residents who had previously communicated their final preferences about death and dying. A HCP commented on how a family member might maintain hope even in the face of advance directives from their spouse:

*What struck me, or resonated with me, was how hard it was for both of them when he [resident] could express some wishes. And even how hard it was for her [family member], even though he had told her to, uh, to stop the ventilator. Withdraw the ventilator. And, the fact that she had hope, until towards the very end. Couple months prior. - HCP*

This comment demonstrates the ethical complexities that can arise when family members are less prepared for death than the resident who has reached the limits of endurance for a technology-dependent life.

The HCPs also noted the distress they experienced by family expectations to be “giving inappropriate care” and the misunderstandings related to perceptions of hastening death. They strongly endorsed the need for formal group discussions between family and HCPs about the outcomes of long-term ventilation and the inevitabilities of pneumonia, severe infections, and other complications that would occur in future. However, according to HCP interviews, some family members were simply unable to process medical information related to disease trajectory due to their own grief and distress. Even though this is an important consideration, and the role and impact of grief needs to be validated, the interviews also indicated the lack of supports and strategies in place for assisting residents and family in understanding the process of disease progression and end-of-life. There was a definitive mismatch between resident and family expectations for care and HCP perceptions about the goals of care.

## Discussion

This research presents a novel and nuanced examination of CCI patient and family perspectives of their health-related expectations in the context of long-term care that is complemented by HCP perspectives. This research is consistent with literature documenting optimistic expectations of families for patient outcomes and discordance with nurse and physician expectations [14, 16, 27]. Yet, in contrast to other literature, there were a number of residents and family who were cognizant of and acknowledged the resident’s poor prognosis, though their accounts also encompassed uncertainty. This uncertainty was a reflection of their past experiences of surviving life-threatening illnesses, including those that HCPs had anticipated they would not survive. In prior research, families demonstrated doubt in the ability of physicians to accurately estimate prognosis [28], and their overly optimistic interpretations appeared to be related to the need to register optimism and their belief that patient characteristics unknown to the physician would lead to better-than-predicted outcomes [29]. Building on this evidence, our research highlights the experiential nature of formulating health-related expectations about an unknown future, which also perhaps mirrors medical uncertainty, particularly considering the unclear and heterogeneous CCI trajectory.

Our research further suggests that the ways in which CCI residents and family make sense of their health and prognosis is not a straight-forward cognitive process of interpreting information. Rather, this is a highly emotional, ongoing and unrelenting process entwined with distress and grief. In our study, conversations about the health-related expectations were difficult, with some becoming sad and tearful and others making efforts to avoid this discussion. An investigation by Nelson and colleagues [30] of CCI surrogate responses to family meetings in the ICU setting described multiple and even antithetical responses that included deflection and rejection, as well as a range of emotions that included anger and grief. Similar responses among the CCI residents and family surrogates in our study suggests that individuals continue to struggle emotionally in the longer term, and also appear to experience prolonged grieving or even complicated grief. It is also possible that chronic critical illness invokes grief over and over at each stage of decline and loss. Our participant narratives suggest that the enormity of psychological symptoms (sadness, worry, distress, and nervousness) reported closer to the time of transition to chronic critical illness [13, 31] likely persists for many. The need to develop and deliver ongoing psychosocial support for both CCI residents and family that accompanies the provision of information and goals of care discussions would perhaps facilitate coping and adjustment and enable residents and family to engage in difficult conversations so that treatment and end-of-life decisions align with resident priorities [32]. Further, others have recommended communication processes and interventions for clinicians to address unmet communication needs common in this population [16, 20, 33]. While further research into the specifics of such interventions is required, existing approaches, such as the Clinical Practice Guidelines for Conducting Family Meetings in Palliative Care that offer a framework for preparing, conducting and evaluating meetings [34], could be adapted.

It is not surprising that the CCI residents and family focussed on the day-to-day challenges more so than longer term prognosis and goals of care considering the enormity of the everyday challenges related to the high symptom burden, full dependency on others and the requirement for life-sustaining therapy. With this in mind, the importance of hope is also unsurprising. The need to maintain hope for something good in one’s life was described by participants as necessary to keep going in the face of what would otherwise seem a purposeless existence. Though not yet discussed in the chronic critical illness literature to our knowledge, this finding is consistent with research with advanced cancer patients, end stage renal disease patients, and patients with other terminal diagnoses, where hope seems to function as a coping mechanism [35–38] and is associated with greater psychological and spiritual wellbeing and the utilization of more effective coping strategies [37]. Sullivan has suggested that hope at the end of life refers to new targets, such as hope for comfort, dignity, intimacy, or salvation, with the challenge being diversifying and redirecting hope rather than protecting or restoring hope [39]. When viewing hope in this manner, hope among CCI individuals is not simply a reflection of unrealistic expectations about prognosis, but a demonstration of active coping and resilience, which if cultivated could be beneficial. Research is needed to explore among CCI individuals and family the use of existing interventions designed to foster hope in other populations, such as The Living with Hope Program [40], Dignity Therapy [41, 42], Life-Review Therapy [43] and the use of a hope conversation guide [44].

The described conflict between some residents/family and HCPs evident in this study as well as HCP discomfort and distress, particularly in relation to end-of-life communication is of particular concern. Leung and colleagues previously identified that nurses caring for CCI patients in the ICU experience internal tension during communication with families in response to their knowledge of the patients’ poor prognosis and anticipation of their death, while simultaneously wanting to shield families from suffering [45]. The complex and demanding work of caring for CCI individuals requires specialized knowledge and skills related to medical care, but of equal importance, the ability to address psychosocial concerns and engage in a therapeutic relationship. HCP efforts to develop trusting and respectful relationships with ventilator-dependent patients are vital to reduce patient uncertainty and discomfort, provide encouragement and support a sense of security, hope, energy and a therapeutic effect [46]. Considering the communication challenges experienced by all CCI ventilated patients and the various use of assistive communication tools, HCPs require greater time and skills to communicate with residents as well as foster therapeutic relationships. However, this is only made possible with appropriate professional and organizational resources, supports and processes of care as well as agreement by the whole care team on priorities.

A palliative approach to care is one that deserves attention in further research with CCI populations. Such an approach emphasizes the importance of therapeutic relationships, and includes the adaption of palliative care knowledge and expertise to individuals, ensuring patient and family needs are addressed early and throughout the illness trajectory, and the integration and contextualization of this approach across healthcare systems [47]. In the absence of both individual and organizational strategies to address the HCP discomfort and distress evident in this study, HCPs caring for the CCI are at risk for work dissatisfaction, disengagement, burnout, and moral distress.

The evidence generated in the qualitative study ought to be viewed with study limitations in mind. Though our findings provide insights that might be relevant to other care environments, other CCI individuals, and other HCPs caring for the CCI, we do not claim that they are generalizable. Study participants were all from one facility designed to provide specialized ventilator care, which might differ from other specialized long term care contexts, not to mention long term acute care hospitals or weaning units. Moreover, the CCI participants were all cognitively intact, English speaking and ventilator dependent but able to communicate. While we included family of CCI patients unable to participate, these were still proxy accounts and family interpretations might not always align with patients themselves. There was a selection bias in that the individuals who self-selected to participate and thus might not represent the full spectrum of perspectives. The strength of this qualitative study lies in the information power rather than the number of study participants, which was enhanced through a relatively narrow study purpose, all participants holding characteristics that were highly specific for the study purpose, and the high quality of the interviews. The interviews lasted much longer than anticipated, participants shared highly emotional, detailed and personal narratives, and there was variation in the perspectives and experiences shared. Furthermore, the input of study team members, inclusive of researchers, clinicians, administrators and patient partners, grounded the analysis and findings in the clinical context and highlighted patient, family and HCP priorities, thereby bolstering clinical relevance as per interpretive description.

## Conclusions

In conclusion, our research offers in-depth descriptions of health-related expectations of CCI patients and family in long-term care, which is further complemented by HCP perspectives. Future research is needed to fully characterize the diversity of experiences and preferences of CCI individuals, and to inform the development and/or adapting of existing communication, psychosocial and health services interventions to ease the tremendous burden experienced by so many patients and families.

## Supplementary Information

**Additional file 1.**

## Data Availability

The datasets generated and analysed during the current study are not publicly available due to the risk of identifying individual study participants, and will not be made available from the corresponding author on request.

## Abbreviations

ICU: Intensive Care Unit
CCI: Chronically Critically Ill
HCPs: Healthcare providers
HCP: Healthcare providers

## References

[1] Nelson JE, Cox CE, Hope AA, Carson SS (2010). Chronic critical illness. Am J Respir Criti Care Med.

[2] Wiencek C, Winkelman C (2010). Chronic critical illness prevalence, profile, and pathophysiology. AACN Adv CritCare.

[3] Chelluri L, Im KA, Belle SH, Schulz R, Rotondi AJ, Donahoe MP (2004). Long-term mortality and quality of life after prolonged mechanical ventilation. Crit Care Med.

[4] Carson SS, Kahn JM, Hough CL, Seeley EJ, White DB, Douglas IS (2012). A multicenter mortality prediction model for patients receiving prolonged mechanical ventilation. Crit Care Med.

[5] Daly BJ, Douglas SL, O’Toole E, Rowbottom J, Hoffer A, Lipson AR (2018). Complexity analysis of decision-making in the critically ill. J Intensive Care Med.

[6] Damuth E, Mitchell JA, Bartock JL, Roberts BW, Trzeciak S (2015). Long-term survival of critically ill patients treated with prolonged mechanical ventilation: a systematic review and meta-analysis. Lancet Respir Med.

[7] Kahn JM, Benson NM, Appleby D, Carson SS, Iwashyna TJ (2010). Long-term acute care hospital utilization after critical illness. JAMA.

[8] Corrado CRN, Ambrosino M, Confalonieri A, Cuvelier M, Elliott M, Ferrer M (2002). Respiratory intermediate care units: a European survey. Europ Respir J.

[9] Lu H-M, Chen L, Wang J-D, Hung M-C, Lin M-S, Yan Y-H (2012). Outcomes of prolonged mechanic ventilation: a discrimination model based on longitudinal health insurance and death certificate data. BMC Health Serv Res.

[10] Douglas S, Daly B, Rudy E, Song R, Dyer MA, Montenegro H (1995). The cost-effectiveness of a special care unit to care for the chronically critically ill. J Nurs Admin.

[11] Roulin M-J, Spirig R (2006). Developing a care program to better know the chronically critically ill. Intensive Crit Care Nurs.

[12] Burns SM, Earven S, Fisher C (2003). Implementation of an institutional program to improve clinical and financial outcomes of mechanically ventilated patients: one-year outcomes and lessons learned. Crit Care Med.

[13] Nelson JE, Meier DE, Litke A, Natale DA, Siegel RE, Morrison RS (2004). The symptom burden of chronic critical illness. Crit Care Med.

[14] Daly BJ, Douglas SL, Lipson AR (2018). Family and nurse prognostication in chronic critical illness. Int J Nurs Res.

[15] Cox CE, Martinu T, Sathy SJ, Clay AS, Chia J, Gray AL (2009). Expectations and outcomes of prolonged mechanical ventilation. Crit Care Med.

[16] Lamas DJ, Owens RL, Nace RN, Massaro AF, Pertsch NJ, Gass J (2017). Opening the door: the experience of chronic critical illness in a long-term acute care hospital. Crit Care Med.

[17] Douglas SL, Daly BJ, Lipson AR (2017). Differences in predictions for survival and expectations for goals of care between physicians and family surrogate decision makers of chronically critically ill adults. Res Rev J Nurs Health Sci.

[18] White DB (2016). Strategies to support surrogate decision makers of patients with chronic critical illness: the search continues. JAMA.

[19] Kahn JM, Carson SS (2013). Generating evidence on best practice in long-term acute care hospitals. JAMA.

[20] Rose L, Istanboulian L, Allum L, Burry L, Dale C, Hart N (2019). Patient and family centered actionable processes of care and performance measures for persistent and chronic critical illness: a systematic review. Crit Care Explorations.

[21] Alberta S.P.O.R. Support unit. Patient engagement in health research: a how-to guide for researchers, 2018. https://albertainnovates.ca/wp-content/uploads/2018/06/How-To-Guide-Researcher-Version-8.0-May-2018.pdf [Accessed 12 January 2020].

[22] Thorne S. Interpretive description: Qualitative research for applied practice. Vol 2. New York: Routledge; 2016.

[23] Lamas DJ, Owens RL, Nace RN, Massaro AF, Pertsch NJ, Moore ST, et al. Conversations about goals and values are feasible and acceptable in long-term acute care hospitals: a pilot study. J Palliat Med. 2017;710–15. 10.1089/jpm.2016.0485 28437209

[24] Charmaz K (2006). Constructing grounded theory: a practical guide through qualitative analysis (introducing qualitative methods series).

[25] Strauss A, Corbin J. Basics of qualitative research: techniques and procedures for developing grounded theory. Thousand Oaks: Sage Publications, Inc; 1998.

[26] Howard F, Crowe S, Haljan G (2019). The chronically critically ill in residential care: healthcare expectations and sources of distress. Crit Care Med.

[27] White DB, Ernecoff N, Buddadhumaruk P, Hong S, Weissfeld L, Curtis JR (2016). Prevalence of and factors related to discordance about prognosis between physicians and surrogate decision makers of critically ill patients. JAMA.

[28] Zier LS, Burack JH, Micco G, Chipman AK, Frank JA, Luce JM (2008). Doubt and belief in physicians' ability to prognosticate during critical illness: the perspective of surrogate decision makers. Crit Care Med.

[29] Zier LS, Sottile PD, Hong SY, Weissfield LA, White DB (2012). Surrogate decision makers' interpretation of prognostic information: a mixed-methods study. Ann Intern Med.

[30] Nelson JE, Hanson LC, Keller KL, Carson SS, Cox CE, Tulsky JA, et al. The voice of surrogate decision makers: family responses to prognostic information in chronic critical illness. Am J Respir Crit Care Med. 2017;864–72. 10.1164/rccm.201701-0201OC PMC5649978 28387538

[31] Jubran A, Lawm G, Kelly J, Duffner LA, Gungor G, Collins EG (2010). Depressive disorders during weaning from prolonged mechanical ventilation. Intens Care Med.

[32] Hickman RL, Douglas SL (2010). Impact of chronic critical illness on the psychological outcomes of family members. AACN Advanc Crit Care.

[33] Moss KO, Douglas SL, Baum E, Daly B (2019). Family surrogate decision-making in chronic critical illness: a qualitative analysis. Crit Care Nurs.

[34] Hudson P, Quinn K, O'Hanlon B, Aranda S (2008). Family meetings in palliative care: multidisciplinary clinical practice guidelines. BMC Palliat Care.

[35] Sachs E, Kolva E, Pessin H, Rosenfeld B, Breitbart W (2013). On sinking and swimming: the dialectic of hope, hopelessness, and acceptance in terminal cancer. Am J Hospice Palliat Med.

[36] McClement SE, Chochinov HM (2008). Hope in advanced cancer patients. Eur J Ca.

[37] Chi GC-H-L (2007). The role of hope in patients with cancer. Oncol Nurs Forum.

[38] Philip J, Gold M, Brand C, Douglass J, Miller B, Sundararajan V (2012). Negotiating hope with chronic obstructive pulmonary disease patients: a qualitative study of patients and healthcare professionals. Intern Med J.

[39] Sullivan MD (2003). Hope and hopelessness at the end of life. Am J Geriatr Psych.

[40] Duggleby WD, Degner L, Williams A, Wright K, Cooper D, Popkin D (2007). Living with hope: initial evaluation of a psychosocial hope intervention for older palliative home care patients. J Pain Symptom Manag.

[41] Chochinov HM, Kristjanson LJ, Breitbart W, McClement S, Hack TF, Hassard T (2011). Effect of dignity therapy on distress and end-of-life experience in terminally ill patients: a randomised controlled trial. Lancet Oncol.

[42] Xiao J, Chow KM, Liu Y, Chan CW (2019). Effects of dignity therapy on dignity, psychological well-being, and quality of life among palliative care cancer patients: a systematic review and meta-analysis. Psycho-Oncol.

[43] Serrano JP, Latorre JM, Ros L, Navarro B, Aguilar MJ, Nieto M (2012). Life review therapy using autobiographical retrieval practice for older adults with clinical depression. Psicothema.

[44] Olsman E, Leget C, Willems D (2015). Palliative care professionals’ evaluations of the feasibility of a hope communication tool: a pilot study. Progress Palliat Care.

[45] Leung D, Blastorah M, Nusdorfer L, Jeffs A, Jung J, Howell D (2017). Nursing patients with chronic critical illness and their families: a qualitative study. Nurs Crit Care.

[46] Tsay SF, Mu PF, Lin S, Wang KWK, Chen YC (2013). The experiences of adult ventilator-dependent patients: a meta-synthesis review. Nurs Health Sci.

[47] Sawatzky R, Porterfield P, Lee J, Dixon D, Lounsbury K, Pesut B (2016). Conceptual foundations of a palliative approach: a knowledge synthesis. BMC Palliat Care.

