# Supplementary material for: Health-related expectations of the chronically critically ill: a multi-perspective qualitative study
Source: BMC Palliat Care. 2021 Jan 4;20:3. doi: 10.1186/s12904-020-00696-w (PMC7781403; doi:10.1186/s12904-020-00696-w)
Supplement: Supplementary file 1 — Additional file 1. [file 12904_2020_696_MOESM1_ESM.docx]

Health-Related Expectations of the Chronically Critically Ill: A Multi-Perspective Qualitative Study

**Interview Guide: Residents**

1. What is your understanding now of where you are with your illness/medical condition?
2. How much information about what is likely to be ahead with your illness would you like to receive from your medical caregivers?
3. How would you describe your quality of life?
4. When you think about the future, what are your expectations for your health?
5. What are your most important goals given your medical condition?
6. What are your biggest fears and worries about your future in regard to your health?
7. What abilities are so critical to your life that you can’t imagine being able to live without them?
8. If you have a setback in your health that makes it harder to reach your goals, how much more would you be willing to go through for the possibility of gaining more time?
9. How much does your family know about your priorities?

**Interview Guide: Family Members**

1. What is your understanding now of where you’re loved one is with their illness/medical condition?
2. How much information about what is likely to be ahead with your loved one’s illness would you like to receive from their medical caregivers?
3. How would you describe the quality of life of your loved one?
4. How would you describe your quality of life?
5. When you think about the future, what are your expectations for your loved one’s health?
6. What are the most important goals given your loved one’s medical condition?
7. What are your biggest fears and worries about the future of your loved one in regard to their health?
8. If your loved one has a setback in their health that makes it harder for them to reach their goals, how much do you think they would be willing to go through for the possibility of gaining more time?

**Interview Guide: Healthcare provider**

1. What is it like for you working at [Facility]?
2. How would you describe the overall quality of care provided to residents?
3. How would you describe resident medical care? What about other types of care provided to residents?
4. What is most important to you in providing care? What does good care look like?
5. What do you think residents and their family members understand of their illness/medical condition?
6. What do you think is important to residents and family members?
7. What do you think are resident and family members biggest fears and worries?
